# Meaning in Life, Subjective Well-Being, Happiness and Coping at Physicians Attending Balint Groups: A Cross-Sectional Study

**DOI:** 10.3390/ijerph18073455

**Published:** 2021-03-26

**Authors:** Ovidiu Popa-Velea, Alexandra Ioana Mihăilescu, Liliana Veronica Diaconescu, Iuliana Raluca Gheorghe, Adela Magdalena Ciobanu

**Affiliations:** 1 Department of Medical Psychology, Faculty of Medicine, University of Medicine and Pharmacy “Carol Davila”, 050474 Bucharest, Romania; alexandra.mihailescu@umfcd.ro (A.I.M.); liliana.diaconescu@umfcd.ro (L.V.D.); 2 Department of Marketing and Medical Technology, Faculty of Medicine, University of Medicine and Pharmacy “Carol Davila”, 050474 Bucharest, Romania; raluca.gheorghe@umfcd.ro; 3 Department of Psychiatry, University of Medicine and Pharmacy “Carol Davila”, 050474 Bucharest, Romania; adela.ciobanu@umfcd.ro; 4 Department of Psychiatry, Clinical Hospital Alexandru Obregia, 041914 Bucharest, Romania

**Keywords:** well-being, meaning of life, happiness, coping, physicians, Balint groups

## Abstract

This study aimed to measure the scores of well-being, subjective happiness, sense of meaning, and coping in Romanian physicians and the potential impact on them of systematically attending Balint groups. Eighty participants (33 men, 47 women, mean age 38.90, SD 9.73) were included in the study. From them, 43 had systematically attended a Balint group in the last two years, while the others represented the controls. All participants were administered the Meaning of Life Questionnaire, the Job-related Affective Well-being Scale, the Brief COPE Scale, and the Subjective Happiness Scale. *t*-tests and MANOVA were used to compare the group scores and the impact of Balint training on the study variables. Results showed that Balintian participants had a lower use of denial and self-blame and were more oriented towards the seeking of emotional and instrumental support. They also reported higher scores in high pleasurable-low arousal emotions, positive emotions, and in the perception of the presence of meaning. Still, when considering other additional predictors (gender, age), the distinct impact of Balint training remained limited to the preference for certain coping mechanisms. These results could stimulate the use of Balint groups as a tool for the physician’s formation programs.

## 1. Introduction

Medicine has become an increasingly challenging profession in recent decades, for a number of reasons, including the rising demands of patients and their families, the significant input of medical technologies, and the ever-growing focus of health care organizations on maximizing their profit [1,2,3]. In this particular context, the physicians’ well-being, happiness, and sense of meaning can be easily challenged and even threatened [4,5,6], while their coping mechanisms are often insufficient to prevent exhaustion and burnout [7]. This is especially true in those medical specialties where the patient’s healing is problematic or the prognosis is poor [8].

So far, the research in this area resulted in abundant, albeit often divergent, outcomes, concerning even the definitions of the study variables. For example, sense of meaning has been widely conceptualized across authors, from “displaying coherence” [9] to possessing “goal directedness” [10], or “a coherent and authentic life narrative” [11]. Previous papers studying the sense of meaning concentrated mainly on its relationship to psychopathology, with substantial evidence linking lack of meaning to distress, depression, anxiety, substance abuse, and suicide [12,13,14,15]. Still, despite these findings with clinical relevance, the presence and the dynamics of the sense of meaning in healthy, theoretically fully functioning individuals—as physicians are presumed to be—have been much less explored.

Concerning well-being in physicians, this has progressively become a topic of interest, not only from the perspective of their personal health, but also because it has tight connections to their ability to provide high-quality patient care, especially concerning patient satisfaction and adherence [16,17]. Previous research has also found a substantial relationship between well-being and the sense of meaning [18,19,20,21], which is generally more sizable for positive well-being dimensions [22]. However, the magnitude of these associations seems to differ, especially in regard to different medical specialties and technologies used in patient care [23]. This, in turn, argues in favor of a better understanding of well-being in physicians and of finding the best ways to measure and to foster it.

Another concept tightly related to well-being and meaning is subjective happiness. This is considered to have registered in the last decades a steady decline, in which health professionals are concerned [24]. Among major contributors to subjective happiness, the sense of personal and professional accomplishment, lack of distress, or the perceived ability to manage workload are often mentioned as critical [25]. To the extent that these parameters are often dysfunctional in physicians, the sense of subjective happiness can remain low for the long term, with no perspectives to be restored, in the absence of energetic stress management interventions.

Regarding the overall ability to confront stress, many coping mechanisms commonly used by physicians are plainly inefficient. As a result, physicians experience a nearly double risk of burnout and work–life dissatisfaction compared to general population, even after controlling for factors such as work hours and level of education [26]. Many health professionals perceive stress as a natural part of their professional life, paying less attention to behaviors aiming its prevention and focusing more on fighting against adversity. From this point of view, in this professional category there is a significant overlap in the definitions of “coping” and “resilience” [27].

As a whole, lacking the sense of meaning, experiencing unsatisfactory well-being, being unhappy, or using dysfunctional coping mechanisms represent reasons of concern, able to influence the physicians’ professional balance and quality of work [18,23]. This legitimizes the increasing endeavors to address these problems through both individual interventions and organizational programs. Among them, the participation in Balint groups has been consistently reported to have a low cost–benefit ratio and to be easy to implement (even in the absence of a solid organizational support). 

During a typical Balint reunion (60–90 min), participants can analyze problematic cases from the perspective of their feelings and behavioral reactions when facing a difficult therapeutic relationship. The group work is typically performed by 6–10 individuals under the coordination of a leader. One of the participants briefly presents a personal case with special emotional significance and brings up some questions and problems to be solved. At the end of the presentation, the other group members may ask for clarification or further information. The presenting individual will then withdraw from the discussion (still, remaining physically present), while the other members debate the case, attempting to find solutions from an empathic perspective and avoiding direct advice, criticism, or reproaches. At the end of the session, the presenting individual symbolically re-enters into the group, in order to express their feedback about what was discussed. A special moment is dedicated for the participants**’** feedback regarding teamwork [28].

In terms of their purpose, Balint groups aim to improve the emotional competence, empathy, and relational skills of physicians and health-allied professions, as well as the prevention of stress-related disorders.

Balint groups have been proved to provide an extensive positive effect on the physicians’ emotional competence, personal awareness, and job satisfaction [29,30,31], offer an ongoing sense of human and professional solidarity to their members, while decreasing perceived isolation and loneliness [32]. Last but not least, Balint groups have been considered to be an efficacious and feasible method of preventing the onset and the progression of burnout [33,34,35,36].

Still, despite these extensive benefits, the impact of Balint training in the medical environment remains largely unexplored and possibly underestimated, partially because of the scattered research on this topic and the diverse methodologies used in the existing publications.

Our study aimed to (1) measure the scores of well-being, the sense of meaning, subjective happiness, and coping mechanisms in physicians and (2) evaluate the distinct impact of the systematic Balint training on these variables. Beyond addressing a gap in the current knowledge in this research area, these objectives indirectly investigate the opportunity of a more systematic use of Balint groups in daily medical practice. Given the availability of Balint groups as a distinct part of formative programs for physicians [37], the identification of a significant influence of Balint training on the sense of meaning, well-being, subjective happiness, and coping could have a substantial importance for building efficient strategies directed towards the preservation of mental health in medical professionals.

## 2. Materials and Methods

### 2.1. Design

The design of the study was cross-sectional, with a single administration of a series of standardized psychometric instruments.

### 2.2. Participants

Physicians under the employment of “Alexandru Obregia” Hospital in Bucharest, Romania, who were between 30 and 65 years old, were considered for this study. Inclusion criteria required them to complete all of the questionnaires and have at least five years of clinical experience. Exclusion criteria were represented by self-reported somatic or psychiatric morbidity, cognitive deficits, other impairments that would render the understanding and completion of the study questionnaires difficult, and lack of completion of one or more study instruments.

A total of 80 physicians were included in the study (33 men, 47 women, mean age 38.90 years old, SD = 9.73, range = 25–63). From them, 43 physicians (“Balintians”—B) had joined and systematically attended, for at least two years, a local Balint group (with at least 6 meetings/year). The other 37 physicians (“non-Balintians”—NB) never attended such a group (or were only occasionally attending one). Their mean work experience was 12.08 years (SD = 9.41, range = 1–35). Furthermore, 17.5% were residents, 56.25% specialists with no PhD degree, and 26.25% specialists with a PhD degree. In terms of marital status, 27.5% were single, 56.25% were married, 12.5% were divorced/separated, and 3.75% were widowers.

The descriptive data of all participants are displayed in Table 1.

### 2.3. Procedure

The study has been run for 6 months, from June–November 2020, and included physicians from the institution who expressed interest to participate and satisfied both the inclusion and the exclusion criteria. The study protocol was designed in accordance with the World Medical Association Declaration of Helsinki and was approved by the Institutional Review Board of the last author’s (AC) institution. Before taking part in this research, all participants completed written informed consent forms, which were distributed to them, together with a brief explanatory statement about the study.

The participants received the study instruments in sealable envelopes, which served for the returned of the completed questionnaires. The exchange of documents was realized in accordance with the general rules of protection against COVID-19 existing in the host institution at the time of the study. A researcher (AM) was available by phone or email, in case there were questions related to the process of filling them in. The responses were processed anonymously, and a numerical code was assigned for each participant. The interpretation of the questionnaires was performed independently by two researchers (LD, AM) and cross-checked for congruence afterwards. Final results were included in an SPSS 21 (SPSS**^®^**, Inc., Chicago, IL, USA) database.

### 2.4. Variables and Instruments

We used the following instruments, which were checked in terms of their validity in the context of the study:

1. The Meaning of Life Questionnaire (MLQ) [38] is a 10-item questionnaire designed to measure two dimensions of meaning in life: (1) “Presence of Meaning” (how much respondents feel their lives have meaning), and (2) “Search for Meaning” (how much respondents strive to find meaning and understanding in their lives). Respondents answer each item on a 7-point Likert-type scale, ranging from 1 (“Absolutely false”) to 7 (“Absolutely true”). MLQ demonstrates good internal consistency, test–retest reliability, and validity [39,40].
2. The Job-related Affective Well-being Scale (JAWS)—short version [41] is a 20-item scale designed to assess people’s emotional reactions to their job. Each item represents an emotion, and respondents are asked how often they have experienced it at work over the prior 30 days. Responses are provided on a five-point scale (“Never”, “Rarely”, “Sometimes”, “Quite often”, and “Extremely often or always”). The JAWS includes a wide variety of emotional experiences, both negative and positive. The emotions can be placed into four categories (subscales), along two dimensions: Pleasurableness and Arousal (intensity). The instrument has been reported to display good validity and reliability (Cronbach alpha = 0.80–0.90) [42,43].
3. The Brief-COPE is a 28-item self-report questionnaire [44], which was developed as a short version of the original 60-item COPE scale [45]. It comprises items that assess the frequency with which a person uses different coping strategies: “Self-distraction”, “Active coping”, “Denial”, “Substance use”, “Use of emotional support”, “Use of instrumental support”, “Behavioral disengagement”, “Venting”, “Positive reframing”, “Planning”, “Humor”, “Acceptance”, “Religion”, and “Self-blame”. Each of the 14 scales is comprised of two items, with each item rated on a 4-point scale, from 1—“I haven’t been doing this at all” to 4—“I’ve been doing this a lot”. The instrument has been described to display good reliability and content validity in studies on health professionals, with an average value of Cronbach alpha coefficient of 0.70 [46,47].
4. The Subjective Happiness Scale (SHS) is a 4-item scale of global subjective happiness. Two items ask respondents to characterize themselves using both absolute ratings and ratings relative to peers, whereas the other two items offer brief descriptions of happy and unhappy individuals and ask respondents the extent to which each characterization describes them [48]. The response format is a 7-point Likert-type scale. A single composite score is computed by averaging the responses to the four items following reverse coding of the fourth item. Scores range from 1 to 7, with higher scores reflecting greater happiness. SHS has high internal consistency and good to excellent reliability and construct validity [49].

### 2.5. Statistical Analysis

Data analysis was performed using SPSS 21 (SPSS**^®^**, Inc., Chicago, IL, USA). The first level of assessment was realized through inventorying the differences between the study groups (Balint and non-Balint attendees) and evaluating their significance (through *t*-tests for independent samples). The associations between systematic Balint training and the sense of meaning, the quality of well-being, subjective happiness, and the choice of coping strategies were analyzed through multivariate analysis of variance (MANOVA). For all these comparisons, the threshold of statistical significance was *p* < 0.05.

## 3. Results

### 3.1. Descriptive Data

The number of participants in the two groups was comparable, with a larger number of women in the non-Balintians group. Still, the two groups did not differ significantly in terms of gender (χ2 (df1) = 2.208, *p* < 0.174). Equally, the groups were similar in regard to age (t (df1) = 1.068, *p* < 0.289), professional qualification (χ2 (df2) = 0.441, *p* < 0.802), work experience (t (df1) = 1.030, *p* < 0.306), and marital status (χ2 (df3) = 0.267, *p* < 0.966) (Table 1).

### 3.2. Scores of the Study Variables and Differences between Groups

The scores of the study variables and the significance of the differences between groups are depicted in Table 2.

The “Presence of meaning” and “Search for meaning” mean scores were situated between 61.15% and 83.32% of the maximal values at the corresponding scales. Averagely, “Presence of meaning” was higher than the “Search for meaning” in both Balintian and non-Balintian participants (ratios B/NB = 1.357 and 1.251, respectively).

Concerning the well-being scores, they were especially higher in Balintian respondents. When assessed by valence and arousal, the mean scores in the sample were also high, with this being especially obvious concerning positive emotions at Balintian respondents, be them accompanied by high arousal or low arousal.

The most preferred coping strategies in the whole sample, but also in the two distinct groups of participants, included, in descending order, active coping, planning, and acceptance, while the least preferred were, in ascending order, denial, substance use, and the use of religion.

In terms of statistically significant differences, the “presence of meaning” was significantly higher in Balintian than in non-Balintian participants (t (df 78) = 2.28, *p* < 0.05). In contrast, “Search for meaning” did not differ significantly between the two groups (t (df 78) = 0.066, ns). These effects were independent by gender.

In what concerns the affective component, the Balintian participants had statistically higher scores in total emotions (t (df 78) = 3.760, *p* < 0.001), positive emotions (t (df 78) = 2.489, *p* < 0.015), and in high pleasurable-low arousal emotions (t (df 78) = 2.573, *p* < 0.012) than the non-Balintians. Regarding the subjective happiness scores, they were placed above 70% from the maximal values indicated by the scale, with Balintian participants scoring slightly higher than non-Balintians (71.92% vs. 70.46%, ns). 

Significant differences between the two groups were met in four coping mechanisms: use of emotional support, use of instrumental support, denial, and self-blame. The use of emotional support and the use of instrumental support were significantly more substantial in Balintian physicians (t (df 78) = 4.624, *p* < 0.001 and t (df 78) = 3.421, *p* < 0.001, respectively). In contrast, denial and self-blame were significantly more used by the non-Balintian physicians (t (df 78) = −4.479, *p* < 0.001 and t (df 78) = −2.333, *p* < 0.022).

### 3.3. The Impact of Balint Training on Meaning, Well-Being and Coping

This effect was investigated through MANOVA, after checking for the multivariate normal distribution of the study variables. In the analysis, we took into consideration both the individual impact of Balint training and its interaction relationships to gender and age. The significant results are synthesized in Table 3.

In terms of coping, Balint training was significantly associated with a higher use of acceptance and a lower use of denial. The use of denial was also partially attributable to the Balint training–age interaction (with lower scores of denial in older individuals), while the ability to positively reframe stressful circumstances was proportional to the respondents’ age, with a partial contribution brought by male gender. Perceived well-being was almost entirely predicted by age (especially in what concerned the ability to experience negative emotions), with a single significant Balint training–age interaction, in regard to the ability to experience LPHA emotions. The dimensions of meaning were not predicted by Balint affiliation or age.

## 4. Discussion

The aims of this study were to assess the sense of meaning, well-being, subjective happiness, and the use of coping strategies in physicians, and offer a measure of the distinct impact of systematic Balint training on these variables. 

When comparing the scores of dependent variables, significant differences occurred between the two studied groups in what concerned the presence of meaning, the ability to experience positive emotions, and the use of certain coping strategies (denial, use of emotional and instrumental support, and self-blame). Well-being scores were higher, as a whole, than the means reported by other authors [50], for both positive and negative emotions. Considering the emotionally charged content of many stressful circumstances specific to the medical profession, these results point out a potential advantage that the systematic Balint training could provide in handling these events. 

Our results offer reasons to assert that this kind of intervention provides an improvement in the ability to better regulate one’s emotions and in avoiding the use of dysfunctional coping strategies, such as denial or self-blame. Participants in the Balint training possessed a higher capability of experiencing emotions, in general, and especially positive emotions with a lower degree of arousal, corresponding to emotional states described under labels such as “at-ease”, “calm”, “satisfied”, or “relaxed” [51]. This type of emotion can have a favorable impact in the long run on their psychological and somatic health [52,53,54], including preventing psychiatric comorbidity and burnout, through mechanisms such as higher affiliation, enjoyment of rewards, and post-goal attainment [55,56,57]. In challenging or stressful circumstances, Balint trainees seem more equipped in finding emotional and instrumental provision, this offering in turn a substantially higher chance to be adapted, through an optimal use of the available environmental resources. They also tend to use acceptance in a productive way, in dealing with unforeseen or unavoidable life circumstances. Part of the characteristic above may be also related to the age- and gender-mediated higher abilities to avoid negative emotions and to positively reframe stressful situations, while, in what concerns denial and the ability to avoid low pleasurable-high arousal emotions (e.g., anxiety, anger), the interaction between Balint training and higher age could represent a possible advantage.

Regarding the sense of meaning, although its presence was significantly higher in Balint trainees (possibly due to the vast number of life experiences that are collected in a Balint group), when considering age as an additional variable, it did not remain significantly correlated to Balint affiliation. Equally, the “search for meaning” proved to be independent from the Balint affiliation and from any other independent variables taken into consideration in this study. This can be due to the presence of other determinants of “search for meaning”, which were not approached in our research. This lack of correlation could be also a reflection of the dual reasons for which individuals seek meaning (possibly because they have not found it, but also because they found it and they need to stop and get a more profound understanding of its nature). In this sense, despite the fact that the medical profession offers countless opportunities to seek meaning (as it often deals with sensitive contents related to suffering and death), the interpretation of meaning remains highly individual, and possibly related to one’s personality characteristics and previous life experience.

Similar to meaning, subjective happiness seems not specifically related to Balint affiliation and almost surely dependent on other predictors, which were not the objective of this research. Variables such as personality type and perceived social support may have influenced not only the relationship between the study variables, but also the self-selection of participants. Additionally, our study has several other limitations, such as the low number of considered variables, its cross-sectional design, the use of self-reporting tools, and the small number of respondents. These limits could be addressed in future research protocols, for example through the development of larger prospective multicentric studies.

## 5. Conclusions

As a whole, our study has foremostly an exploratory intention, to serve as a basis for conceiving new hypotheses about the relationship between Balint training and the physicians’ ability to find meaning, experience well-being and happiness, and cope better. This association could be particularly important in what concerns coping, given the substantial use, in Balint attendees, of behaviors centered on finding and using support, and the rejection of dysfunctional mechanisms of confronting stress circumstances (such as self-blame and denial). The richness of the experienced emotions in a Balint community could also represent, in the long term, an advantage in building better communication skills and express empathy in the therapeutic encounters performed by physicians. Even if these effects seem not to be extended beyond the professional domain to the more profound levels of life meaning or happiness, their positive effect are encouraging. At the organizational level, they could represent a stimulus for the use of Balint groups as a beneficial tool to be included in the physician’s formation programs, throughout their professional pathway.

## Figures and Tables

**Table 1:** Descriptive data of all participants.

Characteristics	Group	Whole Sample	Balint	Non-Balint
Gender	Men (*n*)	33	21	12
	Women (*n*)	47	22	25
Age *		38.900 (9.733)	39.976 (8.892)	37.648 (10.525)
Professional qualification	Residents (*n*)	14	8	6
	Specialists without PhD (*n*)	45	25	20
	Specialists with PhD (*n*)	21	10	11
Work Experience *		12.087 (9.414)	13.093 (8.874)	10.918 (9.001)
Marital status	Single (*n*)	22	12	10
	Married (*n*)	45	24	21
	Divorced/separated (*n*)	10	5	5
	Widowers (*n*)	3	2	1

* mean score (standard deviation).

**Table 2:** Scores of the study variables and differences between groups.

Characteristics		Group	N	Men	Women	t *	*p*
Emotions	Emotions (total)	Whole sample	80	69.150	10.211	-	-
		Balint	43	72.837	8.888	3.760	0.001
		Non-Balint	37	64.864	10.077		
	Positive emotions	Whole sample	80	35.175	6.089	-	-
		Balint	43	36.697	4.306	2.489	0.015
		Non-Balint	37	33.405	7.331		
	Negative emotions	Whole sample	80	24.137	7.231	-	-
		Balint	43	25.395	6.114	1.697	0.094
		Non-Balint	37	22.675	8.188		
	HPHA	Whole sample	80	16.312	3.392	-	-
		Balint	43	16.907	3.014	1.710	0.091
		Non-Balint	37	15.621	3.706		
	HPLA	Whole sample	80	18.862	3.599	-	-
		Balint	43	19.790	2.807	2.573	0.012
		Non-Balint	37	17.783	4.124		
	LPHA	Whole sample	80	12.225	4.015	-	-
		Balint	43	12.976	3.369	1.832	0.071
		Non-Balint	37	11.351	4.547		
	LPLA	Whole sample	80	12.387	4.026	-	-
		Balint	43	12.744	3.922	0.853	0.396
		Non-Balint	37	11.973	4.159		
Meaning	Presence of meaning	Whole sample	80	28.062	4.774	-	-
		Balint	43	29.162	3.578	2.280	0.025
		Non-Balint	37	26.783	5.652		
	Search for meaning	Whole sample	80	21.450	5.595	-	-
		Balint	43	21.488	3.807	0.066	0.948
		Non-Balint	37	21.405	7.197		
Coping strategies	Self-distraction	Whole sample	80	4.400	1.268	-	-
		Balint	43	4.279	1.201	−0.918	0.361
		Non-Balint	37	4.540	1.345		
	Active coping	Whole sample	80	6.775	1.067	-	-
		Balint	43	6.674	0.993	−0.908	0.367
		Non-Balint	37	6.891	1.149		
	Denial	Whole sample	80	2.550	0.761	-	-
		Balint	43	2.232	0.427	−4.479	0.001
		Non-Balint	37	2.918	0.893		
	Substance use	Whole sample	80	2.712	0.766	-	-
		Balint	43	2.744	0.758	0.397	0.693
		Non-Balint	37	2.675	0.783		
	Use of emotional support	Whole sample	80	5.075	1.456	-	-
		Balint	43	5.697	1.058	4.624	0.001
		Non-Balint	37	4.351	1.531		
	Use of instrumental support	Whole sample	80	5.550	1.281	-	-
		Balint	0 43	5.976	1.011	3.421	0.001
		Non-Balint	0 37	5.054	1.393		
	Behavioral disengagement	Whole sample	80	3.012	0.849	-	-
		Balint	43	3.023	0.771	0.121	0.904
		Non-Balint	37	3.000	0.942		
	Venting	Whole sample	80	4.587	1.464	-	-
		Balint	43	4.302	1.282	−1.909	0.060
		Non-Balint	37	4.918	1.605		
	Positive reframing	Whole sample	80	5.337	1.301	-	-
		Balint	43	5.372	1.113	0.255	0.800
		Non-Balint	37	5.297	1.506		
	Planning	Whole sample	80	5.937	1.183	-	-
		Balint	43	6.069	1.055	1.079	0.284
		Non-Balint	37	5.783	1.315		
	Humor	Whole sample	80	4.187	1.159	-	-
		Balint	43	4.279	1.119	0.760	0.450
		Non-Balint	37	4.081	1.210		
	Acceptance	Whole sample	80	6.137	1.198	-	-
		Balint	43	6.279	1.053	1.141	0.257
		Non-Balint	37	5.973	1.343		
	Religion	Whole sample	80	3.475	1.168	-	-
		Balint	43	3.418	0.823	−0.463	0.645
		Non-Balint	37	3.540	1.483		
	Self-blame	Whole sample	80	4.212	1.393	-	-
		Balint	43	3.883	0.980	−2.333	0.022
		Non-Balint	37	4.594	1.690		
Subjective happiness		Whole sample	80	19.950	3.603	-	-
		Balint	43	20.139	3.043	0.505	0.615
		Non-Balint	37	19.729	4.194		

* *t*-test for independent samples, df = 78; HPHA = high pleasurable, high arousal; HPLA = high pleasurable, low arousal; LPHA = low pleasurable, high arousal; LPLA = low pleasurable, low arousal.

**Table 3:** Multivariate analysis of variance (MANOVA) *.

Source	Dependent Variable	Type III Sum of Squares	*df*	Mean Square	*F*	*p*	Observed Power
Balint training	Denial	2.129	1	2.129	4.418	0.031	0.524
	Acceptance	4.565	1	4.565	4.671	0.032	0.542
Age	Emotions (total)	3617.007	31	116.678	2.307	0.023	0.940
	Negative emotions	1793.050	31	57.840	2.202	0.029	0.926
	LPLA	633.867	31	20.447	2.931	0.005	0.983
	Positive reframing	60.689	31	1.958	2.610	0.011	0.967
Balint training x Age	LPHA	188.550	7	26.936	3.149	0.018	0.856
	Denial	6.008	7	0.858	2.490	0.047	0.748
Gender x Age	Positive reframing	23.662	12	1.972	2.629	0.024	0.880

* only statistically significant associations are figured. LPHA = low pleasurable, high arousal; LPLA = low pleasurable, low arousal emotions.

## Data Availability

The dataset presented in this study is available on reasonable request from the corresponding author.
